# Supplementary material for: Regulatory features determine the evolutionary fate of laterally acquired genes in plants
Source: Mol Biol Evol. 2026 Feb 28;43(2):msag042. doi: 10.1093/molbev/msag042 (PMC12949350; doi:10.1093/molbev/msag042)

Supplementary Information for

**Regulatory features determine the evolutionary fate of laterally acquired genes in plants.**

Catherine F Collins, Benjamin T Alston, Samuel GS Hibdige, Pauline Raimondeau, Emily Baker, Graciela Sotelo, Alexander S. T. Papadopulos, Pascal‐Antoine Christin, Lara Pereira, Luke T Dunning

**This Supplementary Information includes:**

Supplementary Figures 1-5

Supplementary Tables 1-8 are in a separated excel file.

Supplementary Figures

**Supplementary Figure 1:** Short read mapping for a donor (*Iseilema vaginiflorum*) and the TPE1-10 *Alloteropsis semialata* accession which lacks this transfer, to the AUS1 reference genome. Coverage plots are shown for three LGT: (a) LGT-084 [ASEM_AUS1_20552], (b) LGT-083 [ASEM_AUS1_20548], and (c) LGT-082 [ASEM_AUS1_20547]. The location of the *cis*-regualatoy elements are indicated in purple


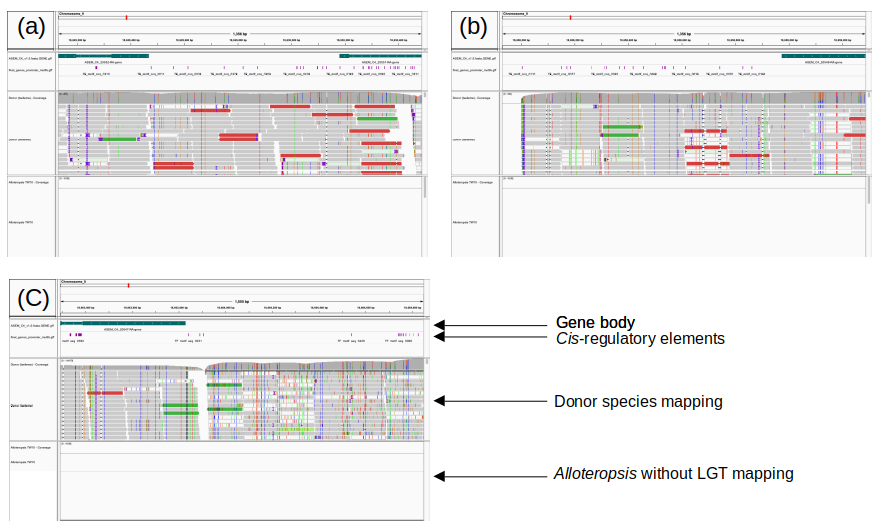


**Supplementary Figure 2**: Expression patterns of LGT-019, phosphoenolpyruvate carboxykinase (PCK). Boxplots show median and interquartile range


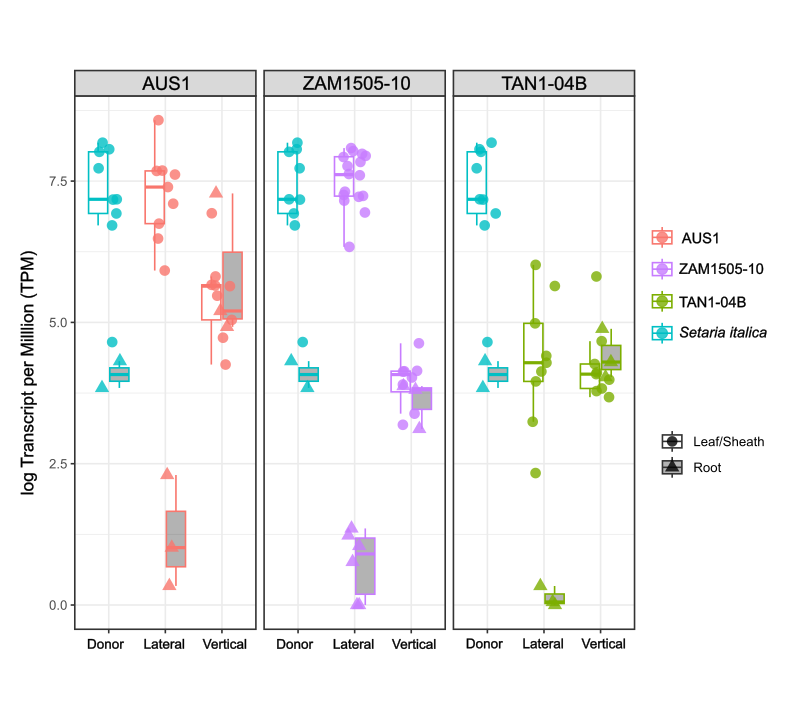


**Supplementary Figure 3:** Genetic divergence between corresponding vertically inherited and donor proxy genes for laterally acquired genes classified as ‘Degenerating’ and ‘Putatively stable’. dS indicates synonymous substitutions between the gene pairs; this value is twice log transformed to create a normal distribution of the data.

**
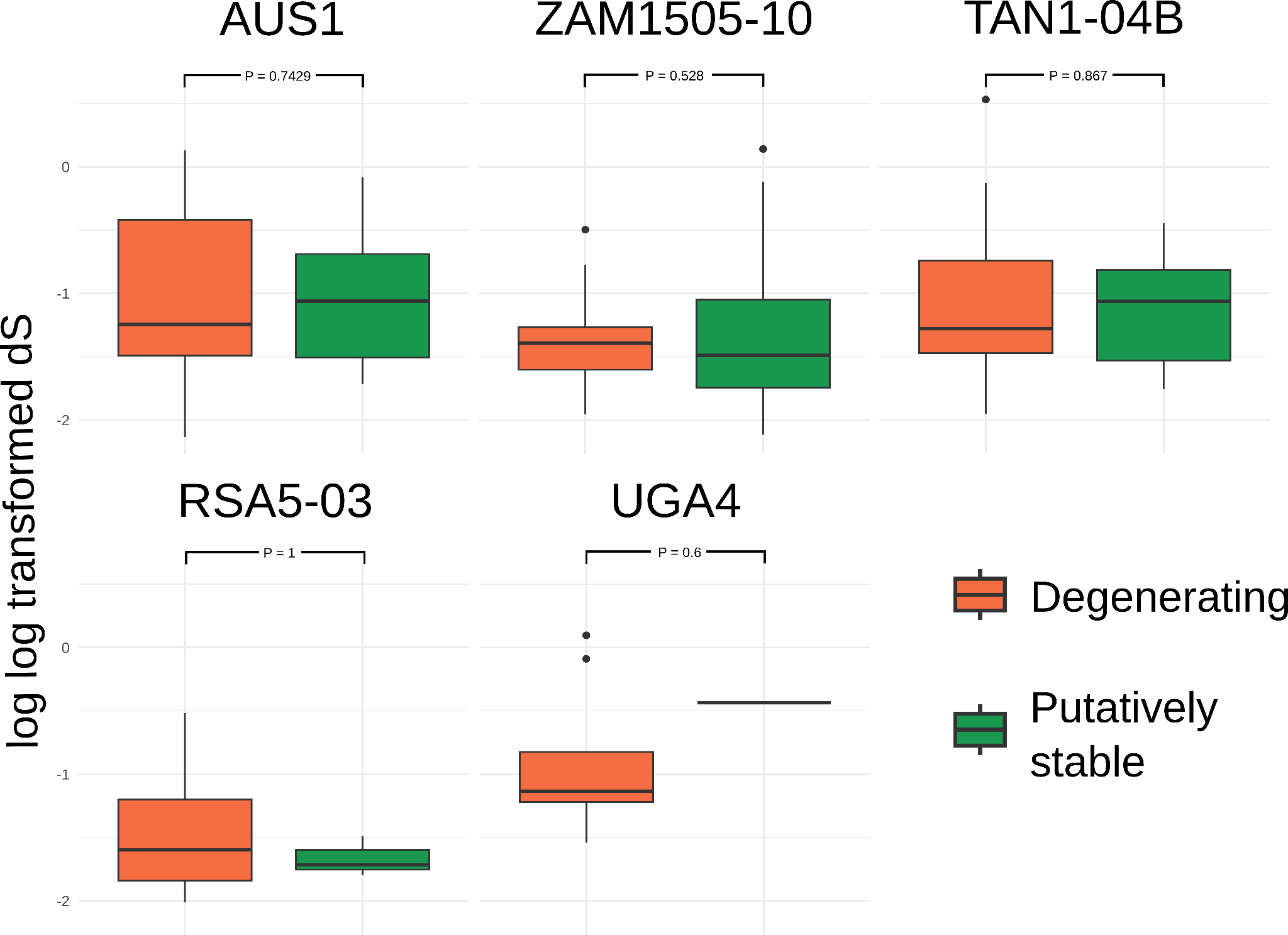
**

**Supplementary Figure 4** - Time since acquisition and expression patterns of laterally acquired genes. A) Relationship between age and expression. Error bars indicate variation across replicates per laterally acquired gene. Black dashed line indicates the linear relationship between age and TPM, with p values from linear mixed effects models included. Coloured lines indicate trends for laterally acquired genes classified as ‘degenerating’ or ‘putatively stable’. No donor indicates laterally acquired genes where a donor proxy gene was not found in the gene phylogeny. B) Boxplots showing the range of molecular age across laterally acquired genes within expression categories. P values from Wilcoxon Signed Rank tests are included above boxplots.

**
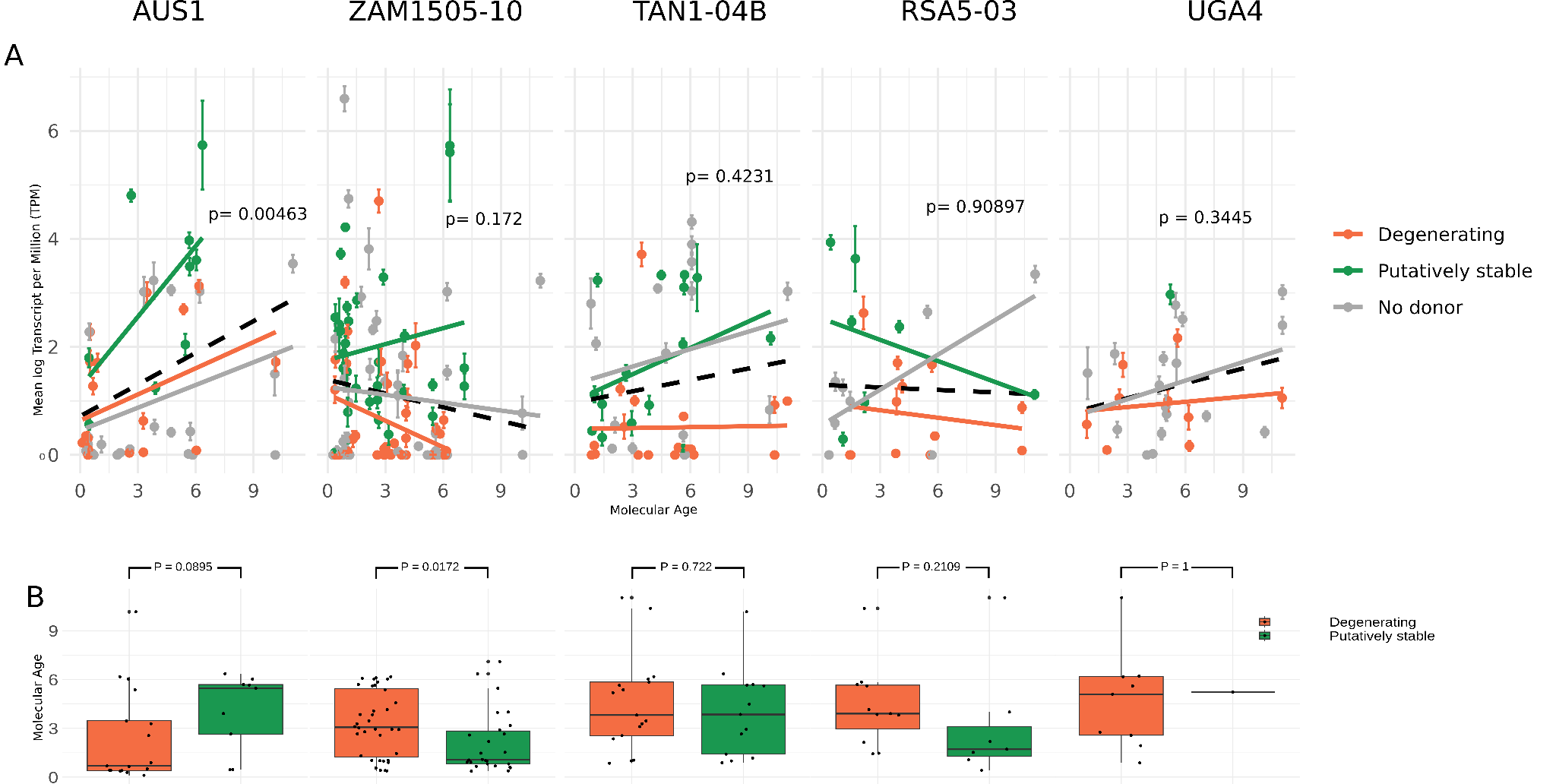
**

**Supplementary Figure 5:** Principal component analysis (PCA) of RNA-seq data demonstrates tissue effect between the leaf and sheath tissue types (red and blue respectively) and the roots (green). Aang = *Alloteropsis angusta*, Asem = *Alloteropsis semialata*, SET = *Setaria italica*, THE = *Themeda triandra*.


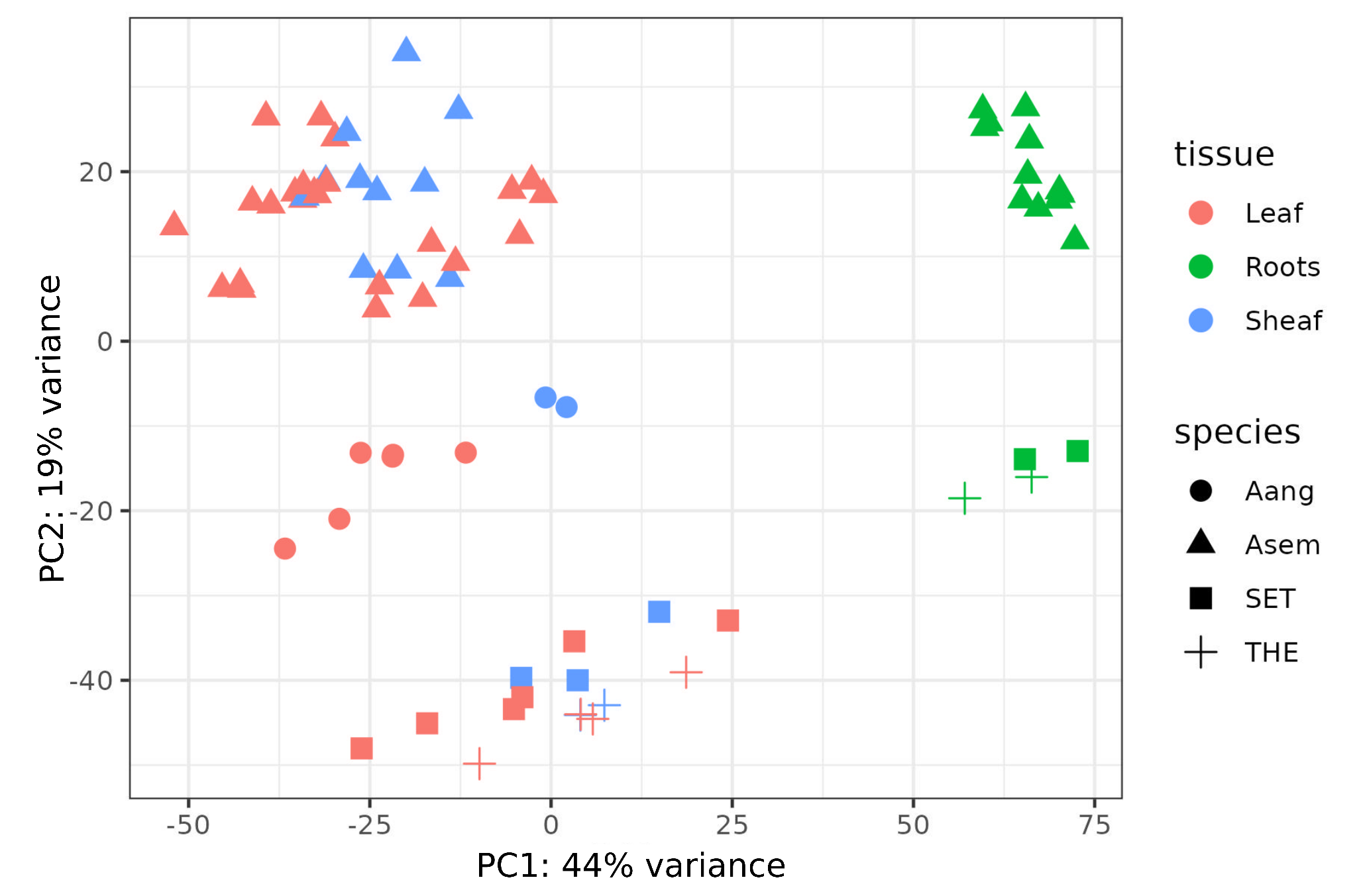

Supplement: msag042_Supplementary_Data [file msag042_supplementary_data.zip › Collins_MBE_SI-v10.0.docx]
